# Toward a Molecular Reclassification of Myalgic Encephalomyelitis/Chronic Fatigue Syndrome: Integrating Multi-Omics, Machine Learning, and Precision Medicine

**DOI:** 10.3390/ijms27104436

**Published:** 2026-05-15

**Authors:** Joshua Frank, Nicole Nesterovitch, Chetana Movva, Nancy G. Klimas, Lubov Nathanson

**Affiliations:** 1 Institute for Neuro-Immune Medicine, Dr. Kiran C. Patel College of Osteopathic Medicine, Nova Southeastern University, Fort Lauderdale, FL 33328, USA; nklimas@nova.edu; 2 Halmos College of Arts and Sciences, Nova Southeastern University, Fort Lauderdale, FL 33328, USA; nn548@mynsu.nova.edu (N.N.); cm3674@mynsu.nova.edu (C.M.)

**Keywords:** ME/CFS, multi-omics, machine learning, precision medicine, post-exertional malaise, biomarkers, transcriptomics, epigenetics, mitochondrial dysfunction, deep learning

## Abstract

Myalgic Encephalomyelitis/Chronic Fatigue Syndrome (ME/CFS) is a complex, multi-system disease characterized by a multitude of symptoms across various organ systems. Diagnosis has relied heavily on heterogeneous clinical symptom presentation and evolving case definitions, with treatment focused on addressing presenting symptoms due to the paucity of validated biomarkers. Meanwhile, advances have been made in understanding the underlying pathophysiology through strong epidemiologic, clinical, and basic science studies. This narrative review synthesizes recent advances that are likely to drive a shift in understanding from symptom-based classification toward a molecularly defined understanding of the disease. This shift in understanding will likely provide the foundation for future research efforts focused on targeting diagnosis and treatment more effectively. Specifically, we reference the identification of rare genetic risk variants through the HEAL2 deep learning framework, the large-scale DecodeME genome-wide association study, and dynamic epigenetic markers of disease state. In addition, the findings revealed the downstream consequences of this genetic and epigenetic priming: chronic innate immune activation, CD8+ T cell exhaustion characterized by upregulation of the exhaustion-driving transcription factors Thymocyte Selection-Associated HMG Box (TOX) and Eomesodermin (EOMES), and a cellular energy crisis centered on mitochondrial dysfunction. Furthermore, results of recent studies have revealed sex-specific transcriptomic and proteomic signatures of maladaptive recovery. We also highlight the role of machine learning and artificial intelligence integrations in translating high-dimensional multi-omics data into actionable biological insights, including the identification of monocyte subsets via Positive Unlabeled Learning, circulating cell-free RNA diagnostic signatures, and integrated multi-modal disease models such as BioMapAI. The combination of these findings, which highlight multiple identifiable mechanisms of molecular activity, support the feasibility of molecular subtyping, precision diagnostics, and targeted therapeutic strategies for ME/CFS.

## 1. Introduction

Myalgic Encephalomyelitis/Chronic Fatigue Syndrome (ME/CFS) is a chronic, complex, and debilitating condition that is characterized by a multitude of symptoms across various organ systems [1]. The constellation of severe, multi-systemic symptoms has been shown to fundamentally disrupt an individual’s activities of daily living, often leaving them unable to work, attend school, or participate in family and social activities [2]. ME/CFS carries a significant financial burden both for patients and the economy, encompassing both direct medical expenses and lost productivity, estimated to be in the tens of billions of dollars in the United States (U.S.) alone [3,4]. Diagnosis has relied heavily on heterogeneous clinical symptom presentation and evolving case definitions, with treatment focused on addressing presenting symptoms due to the paucity of validated biomarkers. Meanwhile, advances have been made in understanding the underlying pathophysiology through strong epidemiologic, clinical, and basic science studies [1]. This narrative review intends to discuss recent advances, serving as a timeline for the progress currently being made from a symptom-based understanding toward a molecular definition. This shift shows promise toward identifying objective biomarkers and targeted treatment more effectively.

### 1.1. Defining a Heterogeneous, Multi-System Disease

Fundamentally, ME/CFS is a systemic illness; it is characterized by dysfunction across the hypothalamic–pituitary–adrenal (HPA) axis, with clinical presentation that is heterogeneous [4]. Significant variations in onset, the specific constellation of symptoms, and overall severity remain prevalent [2,4]. While in some cases the symptomology might be relatively mild, allowing for limited activity, there is a substantial proportion of patients (~25%) that are house- or even bed-bound, requiring extensive care [5]. This variability adds to a further layer of complexity, making this illness even more challenging from a diagnostic and treatment perspective [6,7,8]. In addition, crucial to the disease’s etiology is host susceptibility and varying environmental triggers and how these have interconnected roles in development [1]. Furthermore, it is often reported that over 64% of cases are post-acute infectious episodes. More prevalently in the modern landscape of the disease is a report of 80% or more cases having developed following a diagnosis of Long Coronavirus Disease (COVID)-19 infection [9,10]. There have been implications for other pathogens including Epstein–Barr Virus (EBV), Human Herpes Virus 6 (HHV-6), and enteroviruses serving as triggers [11]. It should be noted that not all instances of these infections result in the development of ME/CFS, which suggests a pre-existing vulnerability [11]. It has also been cited that the disease can be triggered by significant physiological stress such as surgery, physical trauma, or toxic exposures [11].

### 1.2. The Centrality of Post-Exertional Malaise (PEM)

PEM has been widely recognized as a shared and consistent hallmark of ME/CFS [12]. Often described by patients as a “crash,” PEM is a pathological and often delayed flare of symptoms triggered by physical, cognitive, or emotional exertion that far exceeds the effort required by the triggering activity, and that cannot be adequately explained by normal post-exertion fatigue [12]. Throughout this review, we use the term “crash” specifically to refer to this post-exertional symptom flare. It is often misconstrued as a mere increase in tiredness, but PEM has been shown to be associated with a severe exacerbation of presenting symptoms, including but not limited to physical and mental exhaustion, brain fog, muscle and joint pains, sore throat caused by swollen lymph nodes, and even flu-like symptoms [12]. The types of activities that can trigger PEM are wide-ranging and can include minor physical, cognitive, or emotional effort, and the episode may not begin for 24–48 h after the trigger and can last for days to weeks or even longer depending on the specific case [12]. This leads to an obvious and significant reduction in a patient’s capacity to function [12]. Even though PEM prevails as a consistent symptom that can be tracked across different cases of ME/CFS, it is unfortunately subjective, which can be challenging for diagnosis and targeted treatment [12].

In parallel, two-day Cardiopulmonary Exercise Tests (CPETs) have allowed researchers to gain insight into PEM in a controlled laboratory setting [13]. In a recent study, researchers performed a maximal exertion test using a stationary bicycle among patients and health controls (HCs) on a specific day, which was followed by repeating the test the following day. During the test, key physiological parameters were measured with the most notable being oxygen consumption. ME/CFS cohorts exhibit a significant drop in their peak oxygen consumption, otherwise known as VO2 peak or VO2 max [13,14]. This marks a shift in understanding that allows for the collection of biological samples like blood and plasma against the baseline expression of symptoms and at the peak of exertion where PEM inevitably sets. This can in turn be used for the quantification of unique signatures during the stress response [14].

### 1.3. Persistent Challenges in Diagnosis and Patient Stratification

While significant strides have been made through the case definition of ME/CFS, identification of PEM, and objective ways to measure this symptom using CPETs, symptom variability across patients with ME/CFS and the unpredictability of illness progression remain. Without objective biomarkers, diagnosis continues to be complex, involving a process of elimination, which ends up being focused on symptom-based diagnosis and treatment rather than objective biomarkers of disease activity and targeted treatment [1]. This not only makes it difficult for researchers to advance understanding of the underlying pathology but also complicates self-advocacy by patients and challenges physicians’ ability to accurately diagnose the disease [15]. The most used criteria for diagnosis have been the 1994 Fukuda criteria (FC), the 2003 Canadian Consensus Criteria (CCC), and the 2011 International Consensus Criteria (ICC) [8]. Though these criteria have been invaluable in diagnosis, they are not without their faults, namely in the introduction of differing emphasis and strictness in diagnosis criteria, further confounding the phenotypic landscape of the disease through the recruitment of heterogeneous patient cohorts in research studies [8,16,17]. This has often been associated with inconsistent and irreproducible findings. Diagnostic uncertainty, as we have previously alluded to, also impacts patients, as up to 90% of individuals with ME/CFS remain for years of medical consultation before finally being diagnosed [2]. Further confounding diagnoses are the overlapping symptoms with other chronic conditions such as fibromyalgia, postural orthostatic tachycardia syndrome (POTS) and more recently Long COVID [8].

The field of ME/CFS research poses a clear challenge: determining how to diagnose and treat a disease with a wide variability in presentation, diagnosis criteria, and complex symptomology. ME/CFS is not a monolithic single entity, but more likely a large and encompassing constellation of subtypes of chronic illness, each with its own underlying pathology [18]. For instance, patients which exhibit T-cell-driven inflammatory signatures could potentially be grouped separately from those who have a monocyte-driven one [19]. As such, based on this narrative review, we believe that there is an immediate need to turn towards novel methodologies such as omics technologies and machine learning, as well as integrated artificial intelligence (AI) modeling. These methodologies have been invaluable in the advancement of personalized medicine to seek the answers needed to improve diagnosis and target treatment more effectively. By adopting these technologies, the underlying pathophysiology tied to ME/CFS could be elucidated, potentially unveiling unique and quantifiable signatures of the disease activity [20]. The question arises within this narrative review: How have omics technologies and advanced computational methods such as machine learning and AI driven the effort towards a molecular classification of ME/CFS?

## 2. Deciphering the Genetic and Epigenetic Architecture

Research efforts have demonstrated that ME/CFS can cluster in families, which suggests that there is likely a heritable component to the disease [21]. However, the exact underlying genetic architecture has yet to be fully elucidated, and costs coupled with the research population complexities have led to small-scale studies with irreproducible findings [21]. More recently, the advancement of high-throughput sequencing has helped to show that risk is multifactorial and associated with a complex interplay of numerous rare genetic variants, which interact with dynamic epigenetic modifications, resulting in a predisposition to environmental triggers [2,21].

Genome-wide association studies (GWASs) aim to survey millions of common genetic variants at the genome-wide level, which have been helpful in identifying genetic predisposition across complex neuroimmune conditions [22]. However, even with large-scale GWASs utilizing extensive cohorts and revealing critical clues such as variants in the *TPPP* gene, there has been a drawback in the reproducibility of findings [22]. As such, conclusions from GWASs conducted among patients with ME/CFS have not provided the full picture of genomic contributions to disease onset and progression and the role of heritability.

### 2.1. A Paradigm Shift: Deep Learning and the Analysis of Rare Variants

Most recently, a shift has occurred from the study of common variants to rare variants coupled with the application of sophisticated machine learning algorithms [2]. A novel deep learning framework, HEAL, recently advanced to HEAL2 [2,23]. HEAL2 was employed to aid in whole-genome sequencing (WGS) analysis across multiple ME/CFS cohorts [2]. HEAL2 integrates rare coding variants, their predicted functional scores, and their interactions with known protein–protein interaction networks (PPIs) to return a personalized genetic risk score [2]. HEAL2 was able to generate individual risk scores for ME/CFS based on personal rare variants and link genetic risk to specific clinical symptoms such as brain fog and irritable bowel syndrome (IBS) [2]. Researchers have identified 115 high-confidence ME/CFS risk genes, which have been shown to be characterized by an intolerance to loss-of-function mutations. These risk genes were enriched in biological pathways relevant to ME/CFS, including those across the central nervous system (CNS) and in various immune functions and cells [2].

While a specific mutation may differ from patient to patient, these risk genes converge on similar fundamental biological pathways, primarily neurological and immune function. This convergence on shared pathways, despite genetic heterogeneity between individuals, provides a plausible genetic basis for the familial clustering observed in ME/CFS, as families may share overlapping combinations of these rare risk variants. It also offers a framework for understanding the wide variation in symptoms, as patients carrying different subsets of risk genes would be expected to present with differing patterns of neurological, immune, and metabolic dysfunction. For diagnostics, this architecture has a significant implication: the HEAL2 model demonstrated that ME/CFS risk could be predicted from rare variants distributed across the genome (AUROC = 0.67 on an independent cohort), with no single gene dominating the prediction. This suggests that the future of a genetic test for ME/CFS lies not in the search for a single causative variant, but rather in a complex polygenic risk score calculated from the cumulative burden of rare variants across multiple risk genes [2].

### 2.2. The DecodeME Study: A Large-Scale Genetic Investigation into ME/CFS

The genetic landscape of ME/CFS has been limited not only by irreproducible findings and inherent biological complexity, but also to limited sample sizes. The DecodeME study represents a significant effort to uncover the genetic basis of ME/CFS, with a larger sample size, which included 15,579 ME/CFS cases and 259,909 HCs [24]. It should be noted that this study implemented a stringent case definition and required participants to have a formal medical diagnosis and report experiencing PEM. The cohort was primarily female, and most participants reported that their illness began following an infection [24].

While this study remains ongoing, initial analysis revealed eight distinct loci significantly associated with ME/CFS, which have been shown to be associated with disruptions within the immune and nervous system. This is in line with findings from the HEAL2 framework [2,24]. Primarily, these genes are strongly linked to aspects of neurological and immune responses:

- Response to Infection: Loci were identified near genes such as BTN2A2, OLFM4, and RABGAP1L which have been shown to be involved in the responses to a viral or bacterial infection [24]. OLFM4 has been associated with individuals who reported an infection at the onset of their ME/CFS. This suggests that there is a genetic link with the post-infectious trigger of the disease [24].
- Neurological Process and Pain: Loci were identified near the CA10 gene [24]. This region has been shown to share genetic signals with chronic pain, which was reported by over 86% of ME/CFS cases observed in the study [24]. Genes associated with ME/CFS risk were significantly enriched in 13 different brain regions, which supports neurological involvement [24].

A lack of colocalization of genetic signals to depression or anxiety distinguishes ME/CFS from psychiatric conditions, which often lead to misdiagnosis [24]. These initial results support immune and nervous system involvement in the onset and progression of ME/CFS [24].

### 2.3. The Role of Epigenetics: DNA Methylation as a Dynamic Marker of Disease State

Recent research efforts have demonstrated that environmental triggers such as toxic exposures and viral infections likely play a role in the development and progression of ME/CFS [25,26]. Recent studies suggest that epigenetic modifications including disruptions in DNA methylation likely play a role in ME/CFS [27]. Methylation modifications have been shown to occur in or near genes involved in key pathways related to immune function and cellular metabolism, which could explain a link between these changes in ME/CFS [7,19,21,28].

DNA methylation is not a fixed trait. It can vary in response to physiological and environmental factors. This has been shown in a longitudinal study that followed ME/CFS patients over the course of relapse and recovery [29]. The researchers showed that variations in the DNA methylome were directly correlated with the degree of symptom severity [29]. During a relapse (or “crash”), functional changes in DNA methylation occurred in the regulatory regions of the genome that control metabolic, immune, and inflammatory genes [29]. This complex interplay implies that epigenetic changes could represent a molecular “scar” of past triggers and a “real-time” readout of current physiological stress [30]. The predisposing rare variants may cause an individual’s immune system to be more susceptible to dysfunction, but it is the epigenetic response to a trigger such as a viral infection that may lead to a chronic disease state [2,27,29]. The subsequent observation that exercise or stressors can provoke additional epigenetic changes, resulting in PEM tied to ME/CFS, supports this hypothesis [31,32,33]. Although the degree of methylation can vary from patient to patient, they all point to the same dysfunctional physiological pathways, highlighting that the characterization of a patient’s DNA methylation fingerprint could provide a potent and “dynamic” biomarker [31,32,33]. This biomarker may assist in both diagnosis and provide an objective measure of disease activity, a patient’s response to therapeutic interventions, and a molecular assessment of the severity of PEM [34].

## 3. The Dysregulated Immune System: A Core Pathophysiological Feature

Given the common post-infectious development of the disease, as well as immunological symptoms such as sore throat, swollen lymph nodes, and other flu-like symptoms, it has been long established that immune system dysfunction is likely a major contributor to the disease [35,36]. This is supported by multi-omics research, which has highlighted a complex and paradoxical picture of immune system function at the molecular and cellular level [20,37,38,39,40,41,42]. The immune system in ME/CFS would seem to be both chronically activated and dysfunctional or “exhausted” [38]. This situation strongly suggests an immune system trapped in a self-reinforcing cycle of unresolved inflammation and cellular dysfunction, which underlies most of the core symptoms of the disease [35,38].

### 3.1. Chronic Inflammation and Aberrant Cytokine Signaling

A common finding in the deregulation of the immune system in individuals with ME/CFS is a chronic low-grade state of inflammation persisting years after the original trigger [43]. This chronic inflammation is a cell-mediated event that persists without the presence of a pathogen [43]. Some biomarkers of inflammation have also been identified among patients with ME/CFS, including elevated neopterin levels, which are known to be a byproduct of activated monocytes and macrophages [43].

This inflammation is also mediated and represented by abnormalities in cytokine networks, the small protein messengers that coordinate the immune response. Some studies have shown abnormal levels of circulating cytokines in the plasma of ME/CFS patients [43,44]. These research efforts have revealed high levels of pro-inflammatory cytokines including interleukin-1 beta (IL-1β), interleukin-6 (IL-6), and Tumor Necrosis Factor Alpha (TNFα), while others have identified high levels of anti-inflammatory or regulatory cytokines like TGF-β [43,44]. Notably, there have been instances where the levels of certain cytokines have been directly associated with the experience of the patient. For instance, increased plasma levels of the pro-inflammatory cytokines Granulocyte-Monocyte Colony-Stimulating Factor (CSF2) and TNFα have been found to be associated with increased physical impairment and fatigue in ME/CFS patients [45].

### 3.2. Dynamic Transcriptional Architectures: Sex-Specific Responses to Stress

It is vital to note that the transcriptome of ME/CFS is not static or homogeneous [41]. A recent study utilized CPET to induce PEM and, subsequently, RNA-Seq of peripheral blood mononuclear cells (PBMCs) collected at three different time points: baseline, maximal exertion (where PEM sets in), and recovery were analyzed through differential gene expression and pathway enrichment analyses [41]. The data revealed that male ME/CFS patients during the peak of the exercise challenge responded through an instant, aberrant immune mobilization, which is characterized by an unregulated interleukin-12 (IL-12) signaling cascade [41]. This aberrant cascade of cytokine signaling continues through recovery in males [41].

Meanwhile, the most significant sex-specific differences were identified during recovery responses [41]. In the transcriptome of females with ME/CFS, Herpes Simplex Virus type 1 (HSV-1) pathways were most notably enriched, while males exhibited notable enrichment of instant cytokine signaling pathways [41]. This highlights the heterogeneity of PEM in ME/CFS cases, in which women were more likely to present with low-grade immune activation driven by a latent viral reactivation, and males’ responses were dominated by dysregulated cytokine signals [41].

### 3.3. Transcriptomics Reveal Adaptive Immunity Dysfunction: The Exhaustion of CD8+ T Cells

The immune landscape of ME/CFS is highly dysregulated, which highlights the need for advanced high-throughput molecular techniques. Research efforts employing transcriptomics have highlighted a state of cell-mediated immune exhaustion [37,38]. While the innate immune system appears to be constantly activated, single-cell RNA sequencing has revealed that the adaptive immune system (cytotoxic CD8+ T cells) shows signs of dysfunction and exhaustion [38]. CD8+ T cell exhaustion has been shown to play a key role in the immunopathology of ME/CFS, which could be a factor contributing to the state of chronic inflammation [38]. Single-cell sequencing research efforts have provided a better understanding of the disease process from three perspectives:

- Transcriptional Reprogramming: At the single-cell level, CD8+ T cells have been demonstrated to have significant upregulation in key transcription factors, which drive cellular exhaustion, a state in which T cells lose their ability to fight chronic infections due to prolonged stimulation [38]. Among these transcription factors are Thymocyte Selection-Associated HMG Box (TOX) and Eomesodermin (EOMES). TOX is a master regulator of T cell exhaustion that commits cells to a dysfunctional fate through sustained chromatin remodeling of effector-gene loci, while EOMES cooperates to reinforce terminal exhaustion and silence cytotoxic function [38]. Their sustained co-expression in ME/CFS CD8+ T cells is consistent with a state of durable, epigenetically encoded exhaustion rather than transient suppression [38].
- Epigenetic Priming: ME/CFS memory T cells exhibit an altered chromatin landscape. This physical structure of DNA and its associated proteins are characteristic of exhaustion [38]. These “epigenetic scars” make the gene regions associated with effector function less accessible, while making regions associated with inhibitory pathways more accessible, effectively priming the cells for a hypofunctional immune response [38].
- Protein and Functional Changes: Transcriptional and epigenetic changes also manifest at the protein level. Exhausted T cells in ME/CFS patients show increased surface expression of multiple inhibitory receptors, including PD-1 and SLAMF6, which act as repressors of T cell activation [38].

The immune system dysregulation tied to ME/CFS is characterized by both innate immune hyper-activation and hypofunctional responses, which can be driven by immune exhaustion. Meanwhile, this can be explained to be a theory of chronic unresolved antigen exposure [38]. The innate immune system and its dysfunctional monocytes remain in a state of high alert typically in response to a viral infection. As a result, there is a state of chronic inflammation and constant cytokine signaling [38]. This chronic stimulation represents the conditions that drive the adaptive immune system, specifically in CD8+ T cells, driving their functional capacity into an exhausted state [38]. The innate immune system signals for response to infection, while the adaptive immune system is unable to respond due to its exhausted state [38].

### 3.4. Bridging the Periphery and the Central Nervous System: Neural Inflammation

While the molecular signatures discussed up to this point have come almost entirely from peripheral blood, the integration of all the above signals onto the central nervous system (CNS) is the unique hallmark of ME/CFS. Indeed, the HEAL2 framework revealed that there were significant enrichments for risk genes across 13 distinct brain regions, while some of the signals found in the initial findings of the DecodeME study were linked with genes involved in chronic pain pathways, thereby highlighting a neuro-immune genetic signature of the disease and not simply an isolated peripheral immune disorder [2,24]. The elevated levels of pro-inflammatory cytokines in ME/CFS patients’ plasma, such as IL-6, TNFα, and IL-1β, can cross the blood–brain barrier and trigger microglial activation, changes in neurotransmitter metabolism, and sickness behavior syndromes that are similar to the cognitive and neurological symptoms of ME/CFS patients [43,44,45]. When combined with the mitochondrial dysfunction discussed in Section 4, which particularly impacts energy-hungry tissues like the brain, this highlights that ME/CFS’s “brain fog,” poor sleep quality, orthostatic intolerance, and pain syndrome are downstream effects of the same neuro-immune-metabolic program present in peripheral blood [1,20].

## 4. The Cellular Energy Crisis: Metabolic and Mitochondrial Aberrations

Metabolomics and proteomics have been shown to be associated with a cellular energy crisis [46,47]. This dysfunction is associated with mitochondrial function, which powers the cell and results in an observed system-wide hypometabolic response to stress [46,47]. It has been found that the generation of ATP is crucially impaired in ME/CFS metabolism [47]. Specifically, ME/CFS immune cells exhibit significantly lowered mitochondrial coupling efficiency [47]. This means that even when the mitochondria consume oxygen, they fail to convert it effectively into ATP, leading to a state of “uncoupled” respiration where energy is wasted rather than stored [47]. In addition, proteomic analysis revealed a potential molecular mechanism for this deficit tied to dysregulation of the enzymes governing Pyruvate Dehydrogenase (PDH) and Coenzyme A (CoA) metabolism [47]. PDH is a critical enzyme that converts pyruvate into acetyl-CoA, allowing it to enter the mitochondria for the TCA cycle [47]. The alteration of this pathway suggests a metabolic bottleneck, where fuel cannot efficiently enter the mitochondrial engine, forcing cells into a hypometabolic state, despite the presence of available substrates [47]. This proteomic signature aligns with the clinical presentation of severe fatigue, providing a potential molecular explanation for a patient’s inability to sustain energy output.

### Systemic Maladaptation: The Proteomic Signature of Post-Exertional Malaise

Longitudinal proteomics has also revealed a dynamic failure in recovery mechanisms after physical exertion [48]. Dysregulation was observed in the ability of patients with ME/CFS to recover. It is believed that there is a deep proteomic shift that occurs 24 h post exertion, in which patients display a systemic downregulation of adaptive immune responses such as T and B cell signaling, coupled with an inability to activate cell–cell communication networks [48]. This could indicate that rather than a functional and active recovery response, an ME/CFS patient’s system enters a state of immune exhaustion and stress, as was also demonstrated with single-cell transcriptomic studies [37,38]. This further suggests a compensatory mechanism of anaerobic metabolism in response to recovery from exertion [47,48].

Structural proteins like MYBPC1 and MYL3 have been shown to be depleted during this time frame, which also suggests a direct disruption of neuromuscular integrity [48]. In addition, the molecular basis of this disruption in recovery from exertion was found to be highly sex-specific, where female patients show a disordered, proteomic pattern consisting of heightened expression of thousands of proteins, whereas males displayed a hyporesponsive pattern of protein expression during recovery [48]. However, a unifying theme is that these proteomic profiles correlate well with the clinical picture of delayed symptom exacerbation, offering a molecular link to symptoms such as PEM [48].

## 5. The Modern Biomarker Discovery Toolkit: Machine Learning in an Integrated Multi-Omics Approach

Multi-omics studies have generated a wealth of candidate biomarkers and perturbed pathways in ME/CFS. Meanwhile, the biological complexity of these datasets demands more advanced analytical approaches. Machine learning has proven essential in distilling these complex datasets into biologically coherent and clinically relevant signatures. For example, the HEAL2 framework was invaluable in unraveling the rare variant architecture underlying genetic risk in ME/CFS [2].

Single-cell transcriptomics has been instrumental in revealing immune cell dysfunction in ME/CFS, as evidenced by its role in characterizing CD8+ T cell exhaustion The same technology has also been used to interrogate the innate immune compartment, where decades of bulk cytokine data (elevated neopterin, IL-1β, IL-6, TNFα, and CSF2) have implicated chronic monocyte activation but could not resolve whether this reflects uniform dysfunction or a discrete pathological subpopulation [43,45]. A critical biological question therefore remains: does the monocyte dysregulation in ME/CFS represent a global, uniform change across all monocytes, or is it driven by a distinct aberrant subset [37]?

This computational stratification revealed the underlying molecular mechanisms of monocyte pathology in ME/CFS [37]. When diseased cells within the same individual were compared directly to their predicted normal counterparts, the analysis revealed dysregulation of various cytokine receptors and chemokine signaling genes [37]. The most striking result from this analysis was the involvement of the CCL4 gene, which suggests that the diseased subset of monocytes is dysfunctionally primed for tissue infiltration and inflammation [37]. This analysis bridged gene expression data with clinical phenotypic profiles [37]. Notably, ME/CFS patients possess a heterogeneous profile of diseased monocytes alongside regular functional cells, and the exact proportion of diseased to normal cells varies widely between individuals [37]. The percentage of diseased to normal monocytes was strongly correlated with established clinical metrics of disease severity [37]. These metrics included the Multidimensional Fatigue Inventory (MFI)-20 score, overall general health, and the severity of PEM [37].

The insights from intracellular transcriptomics have been extended further through the analysis of circulating cell-free RNA (cfRNA) [49]. These molecules are released into the bloodstream as a functional cellular turnover mechanism [49]. Meanwhile, its value in the quest for biomarker discovery is in the potential for a minimally invasive “liquid biopsy” that could reflect a real-time picture of gene expression, tissue damage, and intracellular signaling at large [49]. Analysis of plasma cfRNA in 93 ME/CFS patients and 75 HCs initially identified 743 differentially expressed transcripts through standard differential abundance analyses [49]. Meanwhile, through extensive computational modeling and feature selection, this high-dimensional dataset was distilled to a highly specific 21-gene subset [49]. This subset was characterized by regulators of MRNA stability and immune response genes such as HNRNPLL and TGFB2 [49].

In addition, a Bayesian deconvolution algorithm (BayesPrism) trained on existing single-cell ME/CFS atlases traced plasma cfRNA back to its unique cellular sources [49]. This deconvolution revealed increased cfRNA expression originating from plasmacytoid dendritic cells, monocytes, and particular T cell populations in ME/CFS patients, which validated the concept of innate immune hyperactivation [49]. Furthermore, an unexpected pathological insight emerged from the diagnostic model: Samples that were misclassified were strongly associated with aberrant proportions of platelet-derived cfRNA [49]. This finding suggests that baseline platelet dysfunction is a confounding biological factor in the disease [49]. Together, these cfRNA analyses have yielded both a functional diagnostic framework and a deeper pathological understanding of ME/CFS immune pathophysiology, including the involvement of innate immune cells and previously unappreciated platelet biology [49].

A prevailing theory in ME/CFS research is the involvement of HERVs, human endogenous retroviruses [40]. These are ancient viral remnants embedded with human DNA, and their expression can serve as a marker for epigenetic dysregulation and viral etiology [40]. However, interpreting the clinical significance of epigenetic reactivation across hundreds of thousands of HERV elements requires advanced computational approaches [40]. By utilizing high-density microarrays to profile over 305,000 HERV elements in the immune cells of ME/CFS and fibromyalgia patients, significant discoveries have been made into the epigenetic regulation of these embedded viral elements [40].

Through clustering and dimensionality reduction analyses, a direct correlation between ME/CFS severity and HERV deregulation was established [40]. Worse clinical symptom scores including physical and mental fatigue were found to be characterized by intense HERV dysregulation among ME/CFS subgroups [40]. In addition, digital cytometry deconvolution through CIBERSORTx revealed that HERV dysregulation was strongly correlated with the depletion of gamma delta T cells and deregulated expansion of resting CD4 memory T cells and plasma cells [40].

The proteomic landscape of ME/CFS has further benefited from machine learning-driven integration of high-dimensional datasets [45]. Supervised learning approaches were applied to integrate information from three distinct biological compartments: mass spectrometry-based plasma proteomics, plasma cytokines, and the cytokine cargo of plasma-derived extracellular vesicles (EVs) [45]. From a pool of 353 protein analytes, a highly predictive 20-protein diagnostic panel was identified that correctly distinguished ME/CFS cases from HCs with an accuracy of 86.1% (AUC = 0.947) [45]. A consensus signature based on seven proteins which included an increased EV IL-2 and CAMP and reduced structural elements including tubulin maintained 79.1% diagnostic accuracy across all models [45].

The translation of this proteomic information goes beyond binary classification to reveal direct correlations between molecular dysregulation and patient symptoms [45]. By integrating these protein profiles with clinical metadata, biological heterogeneity was translated into quantifiable markers of disease severity [45]. For example, increased levels of inflammatory mediators identified by the models, such as Granulocyte–Monocyte Colony-Stimulating Factor (CSF2) and Tumor Necrosis Factor Alpha (TNFα), were shown to be associated with increased physical symptom severity and fatigue experience in patients [45]. These integrated proteomic analyses provide not only highly accurate diagnostic models, but also direct insights into the inflammatory and immune mechanisms underlying ME/CFS morbidity [45].

Metabolomics in ME/CFS has faced a significant challenge in translating findings into a diagnostic test due to the high rate of comorbidities, as ME/CFS frequently co-exists with or presents similarly to other conditions such as irritable bowel syndrome, depression, and migraines [50]. A study utilizing a cohort from the UK Biobank resource contrasted nuclear magnetic resonance (NMR) plasma metabolomes of 1194 ME/CFS patients directly with seven different, homogeneous comorbid groups and HCs [50]. Given the challenge of ascertaining disease-specific metabolic signals from such an enormous degree of clinical heterogeneity, a multi-variable disease score was developed through machine learning [50]. After systematic evaluation of multiple classifiers, a Light Gradient Boosting Machine (LightGBM) model with forward feature selection emerged as the most effective approach [50].

This approach distilled hundreds of data points down to a concise, predictive 28-feature set, which included 19 baseline clinical features and nine specific NMR biomarkers [50]. With this algorithmically defined set, the model was able to detect ME/CFS cases with an Area Under the Curve (AUC) of 0.83 and a recall of 0.70 [50]. This integrated score had a correlation with ME/CFS that was roughly 2.5 times as strong as the best individual metabolic biomarker alone [50]. In addition, explainability analysis through SHAP values revealed that specific metabolic changes, including lower very low-density-lipoprotein (VLDL) particle levels, were among the strongest contributors to the disease signature alongside physical fatigue measures [50].

Collectively, these studies demonstrated that multi-omics integration across genomics, transcriptomics, proteomics, and metabolomics, when coupled with appropriate computational methods, highlights the underlying neuro-immune and metabolic dysfunction associated with ME/CFS. As such, it can be suspected that biological insights that emerge from these analyses would be unattainable through any single omics approach or traditional statistical framework alone.

## 6. The Future of Complex Illness Research: From Machine Learning Tools to AI-Driven Integration

The biological complexity of multi-system chronic disorders such as ME/CFS demands that multi-modal data integration continues to advance beyond individual omics analyses. The distinction between machine learning as a tool for individual data analysis and AI as a framework for synthesizing across these tools is central to building predictive models of disease biology. Moving from isolated classifiers toward integrated AI models of multi-omics data presents a promising path for gaining insight into disease pathology and treating ME/CFS more effectively.

Recently, the development of BioMapAI has represented a shift from diagnostic classifiers and biomarkers to comprehensive disease modeling in ME/CFS [51]. Unlike other studies that we have discussed which might utilize a singular machine learning approach to determine classification based on a singular data type, BioMapAI uses deep architecture designed to consume five unique layers of biological data including gut metagenomics, plasma metabolomics, deep immune profiling, and clinical blood panels [51]. This sophisticated approach to AI does not simply output a binary classification of “diseased” or “non-diseased,” but rather effectively maps the non-linear relationships between the microbiome, the immune system, and the metabolome to predict a complex matrix of 12 unique clinical symptom scores based on the heterogeneous phenotype of the input data from the patient [51].

In addition, this methodology represents a crucial example of the shift to “Explainable AI” (XAI) models, a critical development in terms of clinical utility and trust [51]. By incorporating the SHAP methodology directly into the workflow of the neural network, the model itself can be transparent, enabling the visualization of the “connectivity map” of the disease, thereby identifying the fact that the metabolic cross-talk between the gut and the host, including the tryptophan and butyrate pathways, is not only correlated with the disease state but is, in fact, mechanistically related to particular symptomologies, including pain and fatigue syndromes [51]. Ultimately, the development of BioMapAI exemplifies that the future of complex disease states will be determined by the sophistication of translational approaches and the integration of all these “threads” of understanding into a cohesive, unified approach to the disease state itself [51].

## 7. Discussion: Toward a Molecular Classification of ME/CFS

A potential shift from a symptom-based model of ME/CFS to a molecularly defined landscape is critical for the underlying disease pathophysiology and targeting treatment more effectively. For many decades, the field has been hampered by a lack of reproducibility. Recent efforts into the integration of multi-omics and machine learning techniques have tracked the disease process from its genetic beginnings to its systemic effects. Researchers are beginning to identify a biological convergence.

### 7.1. The Genetic–Epigenetic Interface: Emerging Evidence of a Primed System

Recent data has indicated that the biological basis of ME/CFS includes a genetic predisposition that primes the system for dysfunction [2]. HEAL2 identified 115 high-confidence risk genes, which are enriched in the CNS and the immune system, supporting that ME/CFS is not a monolithic disease [2]. This “neuro-immune axis” has also been supported by the initial data from the DecodeME study, which identified genes that are associated with the response to infection and chronic pain, effectively distinguishing ME/CFS from psychiatric disorders at the molecular level [24].

While genetics explains inheritance and risk, it does not provide an explanation for the dynamic and changing nature of the disease and its symptoms. New studies have indicated that epigenetic factors may hold the key to an ever-changing bridge between genetics and environmental triggers. This may manifest in the form of a molecular memory of past exposures and triggers of the disease, such as viral infections [27]. Studies have shown that changes in the DNA methylome have been associated with the severity of the disease during the process of relapse and recovery [27,30]. This may help to provide an objective way of determining disease states.

### 7.2. Investigating the Core Pathology: Mitochondrial and Immune Dysfunction

This genetic and epigenetic priming can lead to the cellular energy crisis described in recent transcriptomic, proteomic, and metabolomic data [38,46,47]. There is evidence to support a state of chronic low-grade activation, which translates to an exhausted state. While the innate immune system is hyper-activated and pro-inflammatory, the adaptive immune system is exhausted and unable to respond to the inflamed state [37,38].

Single-cell transcriptomics data suggests that CD8+ T cells may be locked in a cycle of exhaustion [38]. In addition, the immunological disorder appears to be mechanistically related to a metabolic bottleneck [46,47]. In this regard, proteomic analysis suggests that there may be dysfunction in ATP production, thereby culminating in clinically observed fatigue driven by an exhausted immune system [38,46,47].

### 7.3. The Molecular Anatomy of PEM and Sex-Specific Divergence

The value of the CPET challenge has been the ability to elicit a “crash” of PEM under controlled conditions, facilitating the exploration of the underlying biological mechanisms of the “crash” [13,14]. As a result, recent transcriptomic studies into the molecular basis of PEM indicate that, despite the similar clinical manifestation of PEM, the underlying mechanisms may be substantially different between the sexes [41].

These results underscore the importance of the fact that, to achieve any meaningful results, future therapeutic trials will likely have to account for the biological differences between the sexes [41].

### 7.4. Validating Pathology: Machine Learning as a Translational Bridge

This neuro-immune and metabolic dysfunction is a complex phenomenon, and omics data being generated can be characterized as high-dimensional, making them hard to interpret using traditional statistical methods. Machine learning has proven to be a vital component for narrowing these broad datasets into potential biomarkers or therapeutic targets [37,40,45,49,50].

- Refining Immune Signatures: By applying Positive Unlabeled Learning to single-cell data, researchers have successfully identified a specific subset of monocytes characterized by the upregulation of CCL4, validating the theory that specific “diseased” cell populations may drive chronic inflammation [37].
- Exploring “Liquid Biopsies”: Advanced algorithms applied to cell-free RNA (cfRNA) have identified a preliminary 21-gene signature, including immune regulators like HNRNPLL and TGFB2, offering a potential non-invasive window into tissue-level immune exhaustion [49].
- Linking Viral Elements: The application of clustering algorithms to human endogenous retrovirus (HERV) expression patterns has provided early evidence linking these ancient viral elements to T cell depletion and clinical severity [40].
- Proteomic Panels: Supervised learning models have identified a panel of 20 proteins, including IL-2 and TNF-α, which correlated with fatigue severity in initial studies [45].

### 7.5. Future Directions: Toward Unified AI Modeling

Though individual machine learning models have successfully been used to validate individual components of the disease, the future of precision medicine in ME/CFS is likely to be through multi-modal integration. Such a trend is exemplified by the BioMapAI framework, which is a promising proof of concept [51]. Through the simultaneous integration of gut metagenomics, metabolomics, and immune system profiling, this model aims to capture the non-linear interactions between these systems. In turn, this model has mechanistically linked gut–host metabolic cross-talk to individual symptom clusters, although more replication and expansion of such models are required to confirm their utility [51].

### 7.6. Toward a Candidate Framework for Molecular Subtypes

A central challenge highlighted throughout this review is that independent cohorts have produced overlapping but not identical molecular findings, in part because each cohort has been stratified only by clinical criteria that themselves capture biological heterogeneity. Rather than treating these divergent findings as inconsistencies, it is likely that they represent different subsets of a disease that should be parsed individually. Based on the convergent evidence discussed above, we theorize that there are four candidate axes along which patients could be stratified in future studies:

- Innate-inflammatory/monocyte-dominant subtype—defined by overexpression of CCL4, diseased monocytes, elevated CSF2 and TNFα, and high MFI-20/PEM severity scores [37,43,45].
- Adaptive-exhausted/CD8+ T cell-dominant subtype—defined by upregulation of TOX and EOMES, elevated surface PD-1 and SLAMF6, and chromatin signatures of terminal exhaustion [38].
- Metabolic-mitochondrial subtype—defined by reduced mitochondrial coupling efficiency, PDH/CoA pathway dysregulation, and a characteristic VLDL-low NMR metabolomic signature [46,47,50].
- Viral-reactivation subtype—defined by HERV activation patterns, HSV-1 pathway enrichment (particularly in female patients), and cfRNA signatures of plasmacytoid dendritic cell and monocyte turnover [39,40,49].

We propose that these are individual patient presentations that are expected to be exhibited separately or in combination, with a sex-stratification layer (given the distinct male IL-12 mobilization versus female HSV-1 reactivation recovery signatures) applied across all subtypes [41,48]. Operationalizing this framework will require coordinated application of multi-omics panels, followed by unsupervised clustering on the integrated feature space. Frameworks such as BioMapAI demonstrate that this is now technically feasible [51].

## 8. Conclusions

The diagnosis and treatment of ME/CFS have relied heavily on symptom presentation, as the underlying mechanisms of disease activity have yet to be fully elucidated. Meanwhile, advances in understanding the underlying pathophysiology through advances in technology and approaches have provided insight into key pathways tied to the onset and progression of ME/CFS. Specifically, currently available research tools including multi-omics approaches, and advanced computation, have aided in some understanding of the underlying molecular mechanisms tied to disease activity. Specifically, research efforts have revealed that genetics may create a predisposed system, epigenetic remodeling may register the history of exposures and triggers, and mitochondrial uncoupling may starve the immune system [2,24,38,46,47].

In addition, the “crash” associated with PEM has begun to be understood not only as a self-reported symptom, but also as a quantifiable failure of molecular recovery, which has the potential to be mediated by different mechanisms for men and women [41]. As the development of these precision medicine approaches and AI tools become more refined, these developments establish the framework required for future targeted therapies based upon the molecular fingerprint of the individual patient.

## Data Availability

No new data were created or analyzed in this study. Data sharing is not applicable to this article.

## Abbreviations

The following abbreviations are used in this manuscript:

ME/CFS	Myalgic Encephalomyelitis/Chronic Fatigue Syndrome
HPA	Hypothalamic–Pituitary–Adrenal
PEM	Post-Exertional Malaise
CPET	Cardiopulmonary Exercise Test
GWAS	Genome-Wide Association Study
WGS	Whole-Genome Sequencing
SNP	Single-Nucleotide Polymorphism
CNS	Central Nervous System
DNA	Deoxyribonucleic Acid
RNA	Ribonucleic Acid
PBMCs	Peripheral Blood Mononuclear Cells
RNAseq	RNA Sequencing
HSV-1	Herpes Simplex Virus Type 1
EBV	Epstein–Barr Virus
NK	Natural Killer
CD8+	Cluster of Differentiation 8-Positive
CD4	Cluster of Differentiation 4
TOX	Thymocyte Selection-Associated HMG Box
EOMES	Eomesodermin
PD-1	Programmed Cell Death Protein 1
ATP	Adenosine Triphosphate
TCA	Tricarboxylic Acid
NMR	Nuclear Magnetic Resonance
cfRNA	Cell-Free Ribonucleic Acid
HERVs	Human Endogenous Retroviruses
EVs	Extracellular Vesicles
IL-2	Interleukin 2
IL-12	Interleukin 12
TNF-α	Tumor Necrosis Factor Alpha
CSF2	Granulocyte–Monocyte Colony-Stimulating Factor
VLDL	Very Low-Density Lipoprotein
SHAP	SHapley Additive exPlanations
XAI	Explainable Artificial Intelligence
AI	Artificial Intelligence
ML	Machine Learning
AUC	Area Under the Curve
LightGBM	Light Gradient Boosting Machine
POTS	Postural Orthostatic Tachycardia Syndrome
MFI	Multidimensional Fatigue Inventory
CCL4	C-C Motif Chemokine Ligand 4
